# Multi-task deep learning network to predict future macrovascular invasion in hepatocellular carcinoma

**DOI:** 10.1016/j.eclinm.2021.101201

**Published:** 2021-12-09

**Authors:** Sirui Fu, Haoran Lai, Meiyan Huang, Qiyang Li, Yao Liu, Jiawei Zhang, Jianwen Huang, Xiumei Chen, Chongyang Duan, Xiaoqun Li, Tao Wang, Xiaofeng He, Jianfeng Yan, Ligong Lu

**Affiliations:** a Zhuhai Interventional Medical Centre, Zhuhai People's Hospital (Zhuhai hospital affiliated to Jinan University), Zhuhai, China; b School of Biomedical Engineering, Southern Medical University, Guangzhou, China; c Guangdong Provincial Key Laboratory of Medical Image Processing, Southern Medical University, Guangzhou, China; d Guangdong Province Engineering Laboratory for Medical Imaging and Diagnostic Technology, Southern Medical University, Guangzhou, China; e Department of Radiology, Shenzhen People's Hospital, Shenzhen, China; f Department of Biostatistics, School of Public Health, Southern Medical University, Guangzhou, China; g Department of Interventional Treatment, Zhongshan City People's Hospital, Zhongshan, China; h Interventional Diagnosis and Treatment Department, Nanfang Hospital, Southern Medical University, Guangzhou, China; i Department of Radiology, Yangjiang People's hospital, Yangjiang, China

**Keywords:** Hepatocellular carcinoma, Macrovascular invasion, Multi-task deep learning, Clinical factors, Radiological characteristics, AUC, AUC areas under curve, BCLC, Barcelona Clinic Liver Cancer, CT, computed tomography, CI, confidence interval, PD, disease progression, HR, hazard ratio, HCC, hepatocellular carcinoma, IDI, integrated discrimination improvement, MTnet, multi-task deep learning neural network, NRI, net reclassification improvement, OS, overall survival, ROC, receiver operating characteristic, TACE, transarterial chemoembolization

## Abstract

**Background:**

Models predicting future macrovascular invasion in hepatocellular carcinoma are constructed to assist timely interventions.

**Methods:**

A total of 366 HCC cases were retrospectively collected from five Chinese hospitals between April 2007 and November 2016: the training dataset comprised 281 patients from four hospitals; the external validation dataset comprised 85 patients from another hospital. Multi-task deep learning network-based models were constructed to predict future macrovascular invasion. The discrimination, calibration, and decision curves were compared to identify the best model. We compared the time to macrovascular invasion and overall survival using the best model and related image heterogeneity scores (H-score). Then, we determined the need for a segmentation subnet or the replacement deep learning algorithm by logistic regression in screening clinical/radiological factors. Finally, an applet was constructed for future application.

**Findings:**

The best model combined clinical/radiological factors and radiomic features. It achieved best discrimination (areas under the curve: 0·877 in the training dataset and 0·836 in the validation dataset), calibration, and decision curve. Its performance was not affected by the treatments and disease stages. The subgroups had statistical significance for time to macrovascular invasion (training: hazard ratio [HR] = 0·073, 95% confidence interval [CI]: 0·032–0·167, *p* < 0·001 and validation: HR = 0·090, 95%CI: 0·022–0·366, *p* < 0·001) and overall survival (training: HR = 0·344, 95%CI: 0·246–0·547, *p* < 0·001 and validation: HR = 0·489, 95%CI: 0·279 – 0·859, *p* = 0·003)*.* Similar results were achieved when the patients were subdivided by the H-score. The subnet for segmentation and end-to-end deep learning algorithms improved the performance of the model.

**Interpretation:**

Our multi-task deep learning network-based model successfully predicted future macrovascular invasion. In high-risk populations, besides the current first-line treatments, more therapies may be explored for macrovascular invasion.

**Research in context**

**Evidence before this study**

We searched Pubmed with the terms “hepatocellular carcinoma” and “macrovascular invasion”) for papers published between Jan 1, 2009, and July 17, 2021, with no language restriction. We found that most studies focus on the treatments or diagnosis of existed macrovascular invasion, or prediction of microvascular rather than macrovascular invasion. Since treatments for macrovascular invasion should be performed as early as possible, we constructed a deep learning base model to predict the risk of macrovascular invasion.

**Added value of this study**

Our multi-task deep learning network-based model combined clinical/radiological factors and radiomic features, which outperformed clinical model by discrimination and calibration in both datasets. Subdivided by the model itself and its related image heterogeneity scores, the high-risk subgroups had significant short median time to macrovascular invasion and overall survival. Furthermore, an applet was constructed for further clinical application.

**Implications of all the available evidence**

Our combined model could predict the risk of future macrovascular invasion in hepatocellular carcinoma. For patients with high risks, current guide recommended treatments are not enough in preventing extrahepatic metastasis and macrovascular invasion, systematic treatments such as targeted or immune therapies should be explored.

Alt-text: Unlabelled box

## 1 Introduction

Generally, primary liver cancer ranks higher in mortality (second in men and fifth in women) than in incidence (fifth in men and ninth in women), making it a highly malignant cancer [1]. Pathologically, 70−85% of primary liver cancers are hepatocellular carcinomas (HCCs).[1] For patients without extrahepatic metastasis or macrovascular invasion, liver resection and transarterial chemoembolization (TACE) are the guideline-recommended first-line treatments [2], [3]; however, because HCC is highly invasive and spreads easily into the vascular system, 5.4–38% of treated patients are found to have macrovascular invasion during follow-ups [4]. In contrast to microvascular invasion (which only influences post-resection occurrence), macrovascular invasion can lead to aggressive liver dysfunction and preclude patients from further treatments. Therefore, without proper treatment, the median overall survival (OS) has been reported as approximately 2·7 to 4·0 months [2,4]. Several interventions such as targeted, immune, and radiation therapies can prolong the median OS to over one year [4], [5], [6]. However, the safety and efficacy of such treatments are highly dependent on early diagnosis and treatments [7], [8], [9]. With prediction of future macrovascular invasion, at least we may have two methods to treat macrovascular invasion more effectively: the first is combining immunotherapy when macrovascular invasion is radiologically occult [9]; the second is performing more frequent follow-ups to detect it before it is too late. However, it is evident that strategy may be more reasonable in high-risk population rather than in all the patients. Thus, identifying patients at high risks of macrovascular invasion is the first challenge to be solved before exploring such “early intervention” strategy. Therefore, determination of timely interventions for macrovascular invasion has become a critical clinical challenge in HCC.

The need for early diagnosis and treatment is unique to HCC; thus, experience may be learned from other cancers. In nasopharyngeal carcinoma, to treat distant metastasis more effectively, researchers established a prediction model for future metastasis, which could be used to select high-risk populations for early adjuvant chemotherapy [10]. In a similar manner, prediction models may also help us to establish a warning system for early intervention of macrovascular invasion after liver resection, TACE, or ablation. Although many studies have focused on the prediction of microvascular invasion [11], [12]. we still lack models for macrovascular invasion. The few related studies involve limitations such as combining patients with micro- and macro-vascular invasions (only 18% patients had macrovascular invasion) [13] in the study or only identifying factors regarding existing rather than future macrovascular invasion [14]. Therefore, further exploration is necessary to fill the gap between clinical needs and current studies.

In this regard, deep learning can be informative and helpful. By in-depth mining and efficient analysis of data both within and beyond the traditional visual system, deep learning algorithms bring medicine to the data-driven era [15]. Considering hepatology, deep learning has outperformed the traditional shear wave elastography in assessing liver fibrosis [16]. Researchers have also proven that deep learning outperforms conventional machine learning models in differentiating HCC from cirrhotic parenchyma [17]. Moreover, it has promising performance in predicting disease progression and the OS of HCC [18]. Nevertheless, overfitting is a common problem in deep learning algorithms. Multi-task learning was introduced to control overfitting. Achieving positive feedbacks among related tasks can enrich information and increase the accuracy of each task; thereby, improving the performance the overall model [19]. Therefore, we constructed a multi-task deep learning neural network (MTnet) to construct models to predict macrovascular invasion and assist in early intervention.

Considering the threat of macrovascular invasion and the advantages of MTnet, we conducted a multicenter study to establish and validate the MTnet-based model. Assisted by our model, early intervention can be explored in high-risk populations to achieve additional survival benefits after guideline-recommended treatments.

## 2 Methods

### 2.1 Patients

**Fig. 1:**
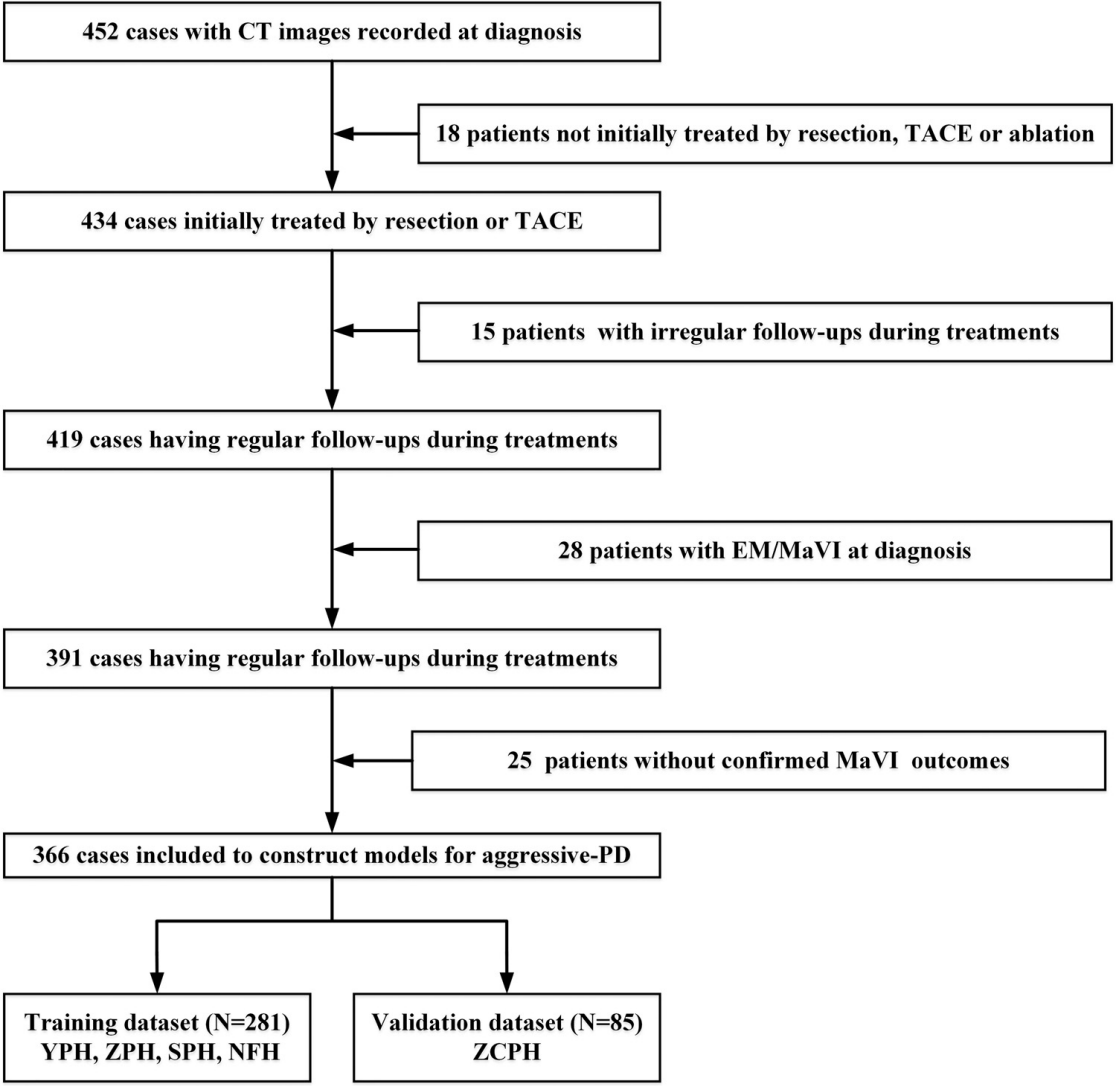
Inclusion and exclusion flowchart of this study.

Data on patients diagnosed with HCC between April 2007 and November 2016 were collected from five Chinese hospitals: Yangjiang People's Hospital (YPH), Zhongshan City People's Hospital (ZCPH), Zhuhai People's Hospital (ZPH), Shenzhen People's Hospital (SPH), and Nanfang Hospital (NFH). Follow-ups were conducted until December 2019. The inclusion criteria were: 1) patients diagnosed with HCC clinically or pathologically; 2) patients with computed tomography (CT) recorded at the time of diagnosis; 3) patients undergoing an initial treatment with liver resection, TACE, or ablation recommended by guidelines of American Association for the Study of Liver Diseases and European Association for the Study of the Liver [2], [3]. 4) patients without extrahepatic metastasis or macrovascular invasion at the time of diagnosis; 5) patients that developed macrovascular invasion after initial treatment, or patients with no subsequent macrovascular invasion for at least one year unless death occurred. By contrast, the exclusion criteria were: 1) irregular follow-ups and 2) presence of other cancers. A flowchart of the patient selection process is presented in Figure 1. Thereafter, the patients were classified into stages, according to the modified Barcelona Clinic Liver Cancer (BCLC) staging system based on the latest guidelines (0, A, and B) [2], [3].

The study protocols were approved by the Ethics Review Committee of the Zhuhai People's Hospital. Informed consent for medical research was waived because patient data were collected retrospectively. All patient data were anonymized before analysis.

### 2.2 Patient and public involvement

Patient and public involvement Patients and/or the public were not involved in the design, conduct, reporting or dissemination plans of this research.

### 2.3 Treatments and follow-ups

The initial treatment options for liver resection, TACE, and ablation were determined by multi-disciplinary teams according to the guidelines [[2], [3],^20^] (tumor characteristics, liver function, and patients’ will). After the initial treatments, regular follow-ups were arranged every four to six weeks for the first year. Subsequently, the interval was doubled every year if the patients had no disease progression (PD). After three years, the interval was stabilized to three months if the patients had no PD. During every follow-up, chest X-rays, abdominal CT or MRI, and necessary laboratory tests were performed. When symptoms of extrahepatic metastasis were identified, an additional CT or MRI for the suspected region was acquired.

### 2.4 Outcomes

The primary outcome of this study was macrovascular invasion during follow-ups, which was confirmed by two radiologists (Y.L. and J.Y.) with 10 years of work experience. Whenever disagreements between Y.L. and J.Y. occurred, a third radiologist (L.L. with over 20 years of working experience) was asked to perform another independent assessment. Then macrovascular invasion was finally confirmed by at least two of the three radiologists. Macrovascular invasion was determined based on the enhancement of arterial phase imaging, expanding vessel, and/or direct extension into the vasculature [21]. The secondary outcome was OS, which was calculated from the initial treatments to the date of death.

### 2.5 Candidate clinical factors and radiological characteristics

The clinical factors included patients’ status (age, sex, Child-Pugh grade, HBV infection, and CT identified cirrhosis); tumor burden (location, lesion number, maximum diameters, alpha fetoprotein level, and BCLC stages); and initial treatments. Considering the potential value of the qualitative radiological characteristics, we added nine qualitative radiological characteristics as previously reported: [22] fusion lesions, invasive shape, HCC capsule, HCC capsule breakthrough, corona enhancement, corona with low attenuation, mosaic architecture, nodule-in-nodule architecture, and enhancement ratio of the HCC lesions. All these radiological characteristics were assessed by two radiologists (Y.L. and J.Y., both have a working experience of over 10 years) independently. Whenever disagreements between Y.L. and J.Y. occurred, a third radiologist (L.L. with over 20 years of working experience) was asked to perform another independent assessment. Then the final results were decided by at least two among the three radiologists.

### 2.6 CT acquisition and tumor segmentation

The CT parameters for the participating hospitals are listed in Supplementary Table S1. Because the HCC capsule can be visualized more clearly in the portal phase than in the arterial phase [23], to ensure segmentation accuracy, we used the portal phase for the MTnet. Where multiple lesions were present, we identified the target lesion according to the modified response evaluation criteria in solid tumors assessment depending on the longest diameter and suitability [24].

### 2.7 MTnet-based model's construction

**Fig. 2:**
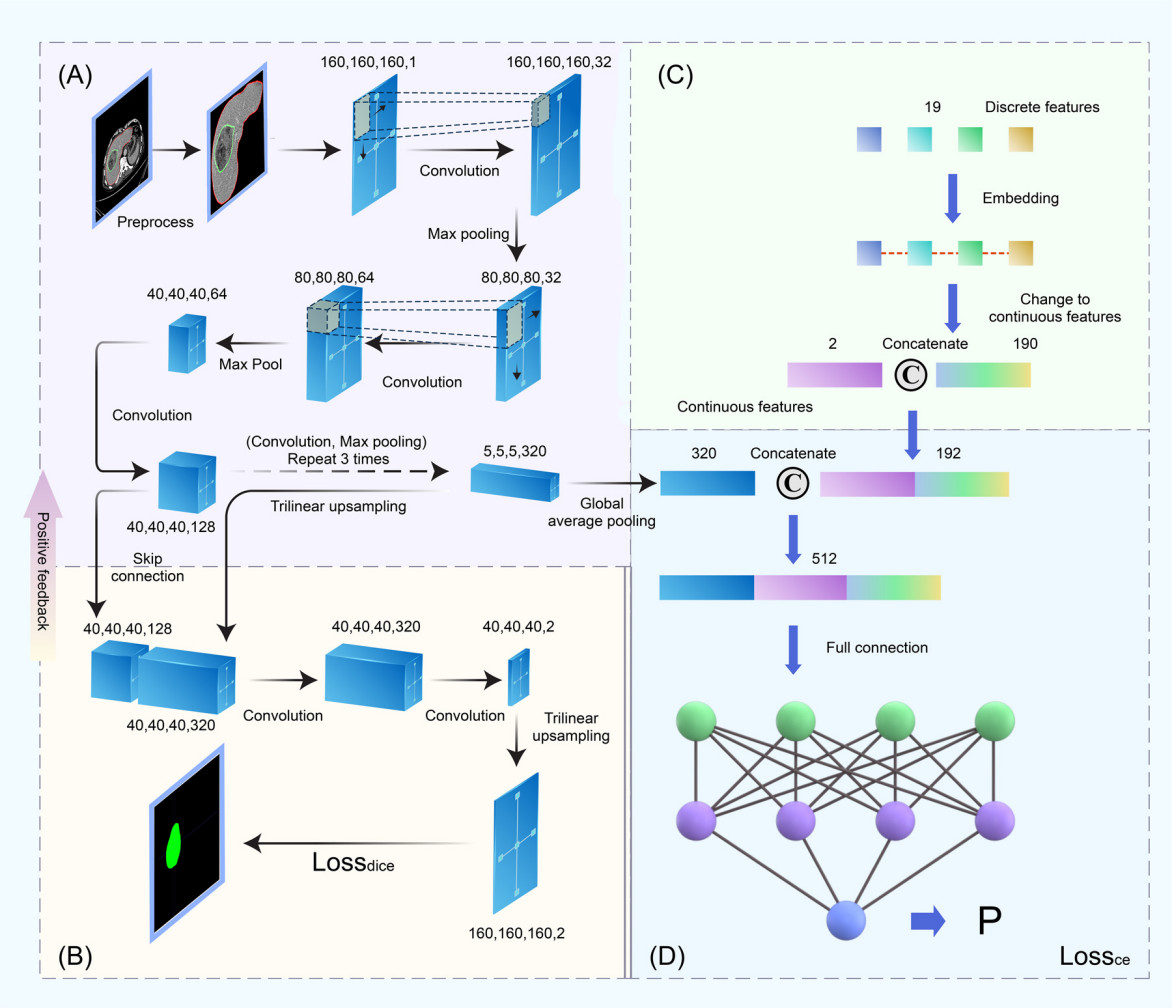
Illustration of the proposed MTnet. (A) Encoder and (B) decoder of the segmentation network, where the decoder was used as a positive feedback to the encoder for extraction of rich image information; (C) Information on clinical and radiological data extracted using two fully connected layers; (D) Combination of image, clinical, and radiological information for macrovascular invasion prediction.

The MTnet consists of two subnetworks: one for segmentation (a modified UNet as shown in Figures 2-A and 2-B) and the other for classification (Figures 2-A, 2-C, and 2-D).

After a manual segmentation of the HCC lesion and an automatic segmentation of the liver, we used three blocks for MTnet. The first block extracted information from the CT images (Figure 2-A and 2-B, detailed in Supplementary Text S1). The extracted information was separated into two flows: one entering process for the tumor segmentation (Figure 2-B), with a positive feedback from tumor segmentation returning to figure 2-A to refine the image information; and the other entering process for the third block (Figure 2-D). The second block extracted information from clinical factors and radiological characteristics (Figure 2-C, detailed in Supplementary Text S2, Text S4). The third block predicted the risk of macrovascular invasion (Figure 2-D, detailed in Supplementary Text S3, Text S4). For the deep learning radiomics model (Model^DR^), information came from the first block. Similarly, information of clinical and radiological data was extracted by the second block to construct Model^CR^. The combined model (Model^CR-DR^) consisted of the entire process of the three blocks.

To test the rationality of the MTnet, we first deleted the subnet of tumor segmentation from the MTnet and constructed a new combined model (Model^NO Seg-CR-DR^). We compared Model^CR-DR^ with Model^NO Seg-CR-DR^ to test whether the subnet for segmentation was necessary. Next, we screened for clinical and radiological factors using conventional logistic regression. We used the identified factors as the input of the aforementioned second block with the deep learning algorithm (Model^Logistic-CR-DR^). We compared Model^CR-DR^ with Model^Logistic-CR-DR^ to test whether the end-to-end deep learning algorithm truly outperformed the conventional logistic regression.

### 2.8 Statistical analysis

The baseline characteristics between the training and validation datasets were compared according to their categorization and distribution. For continuous variables, Kolmogorov–Smirnov test was used to test the normality, student's t-test was performed when they were normally distributed, Wilcoxon rank sum test was performed when they were not normally distributed. For grade variables, Wilcoxon rank sum test was performed. For categorical variables, Pearson's χ2 test or Fisher's exact test were performed.

**Fig. 3:**
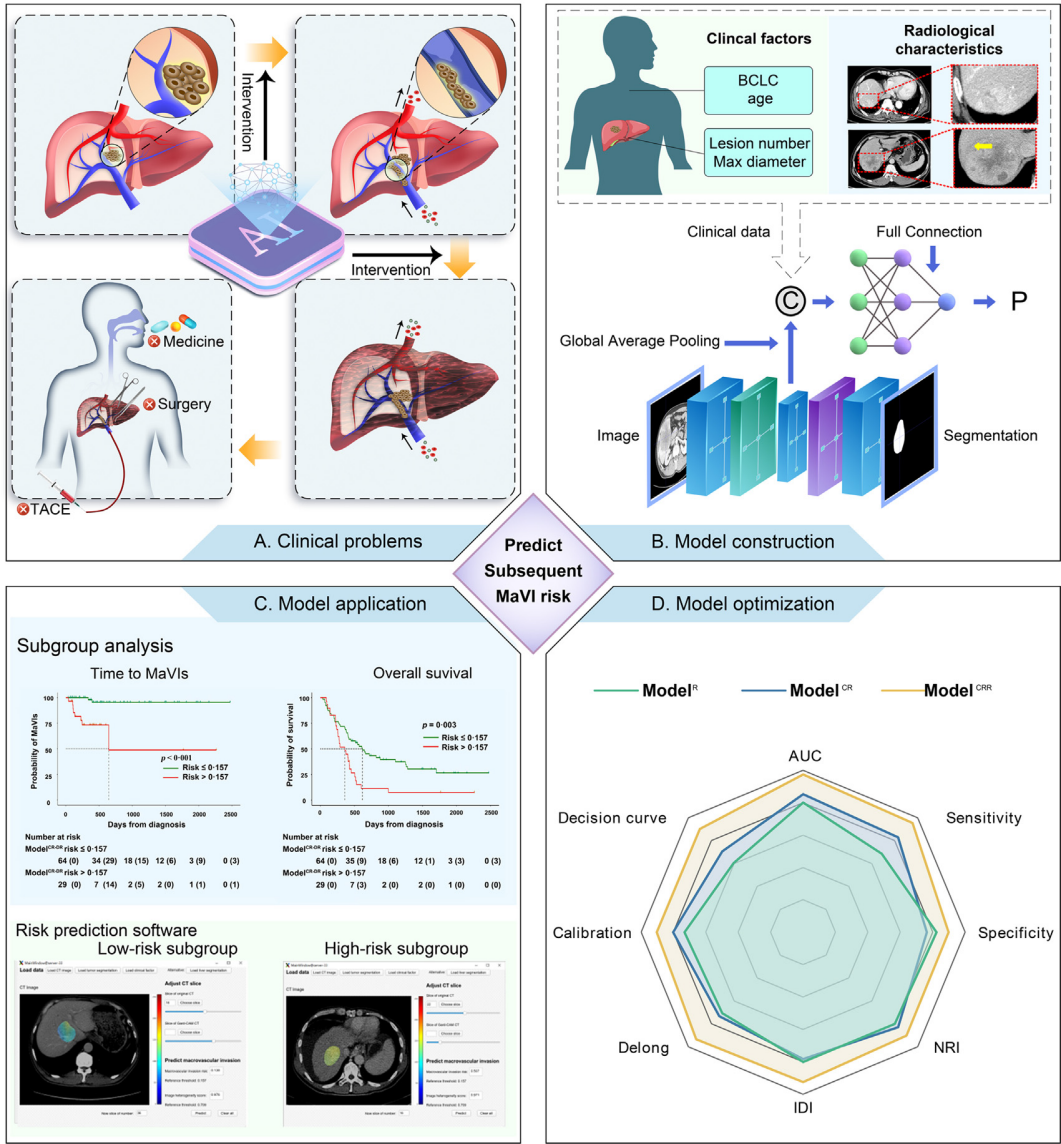
Study design. Without timely intervention, macrovascular invasion causes rapid deterioration of liver function. This disqualified some patients from receiving further treatments (A). We combined clinical factors, radiological characteristics, and radiomics using a deep learning algorithm to construct models (B). Assessed by multiple parameters such as AUC, calibration, and decision curve, we identified the best model (C). We compared the time to macrovascular invasion and overall survival based on the best model. We constructed an applet for the best model (D). AUC: area under the curve; BCLC: Barcelona Clinic Liver Cancer (staging system); IRI: integrated discrimination improvement; NRI: net reclassification improvement; TACE: transarterial chemoembolization.

As noted above, we constructed a radiomic model (Model^DR^) using the first block, a clinical/radiological model (Model^CR^) using the second block, and a combined model (Model^CR-DR^) using the third block. First, to evaluate the models, we compared their discrimination based on the receiver operating characteristic (ROC), using indices such as areas under curve (AUC), Delong test, incorporating net reclassification improvement (NRI), and integrated discrimination improvement (IDI). In addition, we compared their calibration and decision curves. We used the results in the validation dataset to identify the best model. When the difference was limited, we then referred to the results of training dataset. Second, we tested whether the performance of the identified best model was influenced by treatments or the BCLC stages. Finally, to identify the threshold for high-risk populations, we used the Yorden index in the ROC curve to subgroup the patients and compared the time to macrovascular invasion and OS, using Kaplan–Meier plots and the log-rank test. Moreover, to test whether the heterogeneity identified by our MTnet was related to the outcomes, we grouped the patients using the image heterogeneity score within the tumor area (Supplementary Text S5, Supplementary Figure S1) and compared their macrovascular invasion and OS. The overall design of this study adhered to RECORD guideline (Figure 3).

Two-sided *p*-values less than 0.05 were considered as statistically significant. All the analyses were performed using the R and Python software.

### 2.9 Role of the funding source

The funding sources were not involved in the study design, collection, analysis, interpretation of data, writing of the report, or decision to submit the paper for publication.

## 3 Results

### 3.1 Study population and baseline characteristics

A total of 366 patients were included: patients from YPH, ZPH, SPH, and NFH were used as the training dataset (N = 281), whereas patients from ZCPH were used as the external validation dataset (N = 85). During the follow-ups, 45 patients had macrovascular invasion (35 in the training dataset and ten in the validation dataset). Excluding age, initial treatments, and maximum diameter of HCC lesions, there were no statistical differences between the training and validation datasets (Table 1).

**Table 1:** Baseline demographics of patients included in the study.

	Training dataset (N = 281)	Validation dataset (N = 85)	*p*-value
**Age**	55·1±12·0	60·6±12·0	<0.001*
**Sex**			0.512
Male	235	68	
Female	46	17	
**Initial treatment**			<0.001*
Liver resection	79	18	
TACE	196	52	
Ablation	6	15	
**HBV infection (*N*)**			0.315
Negative	21	3	
Positive	260	82	
**Cirrhosis**^1^			0.407
Negative	120	32	
Positive	161	53	
**Child-Pugh class (*N*)**			0.369
A	200	85	
B	36	11	
**BCLC stage**			0.630
0	32	1	
A	169	64	
B	80	20	
**Max diameter (mm)**	61·9 (7·0–198·0)	67·0 (10·0–176·0)	0.005*
**Number of lesions**			0.110
1	188	66	
2	46	7	
3	22	3	
>3	25	9	
**AFP level (ng/mL, *N*)**			0.157
<25	120	31	
25–400	80	22	
>400	81	32	

AFP: alpha fetoprotein; BCLC: Barcelona Clinic Liver Cancer; HBV: hepatitis B virus; TACE: transcatheter arterial chemoembolization.

1 Refers to cirrhosis exhibiting morphological changes in the computed tomography.

### 3.2 Comparison among Model^DR^, Model^CR^, and Model^CR-DR^

**Fig. 4:**
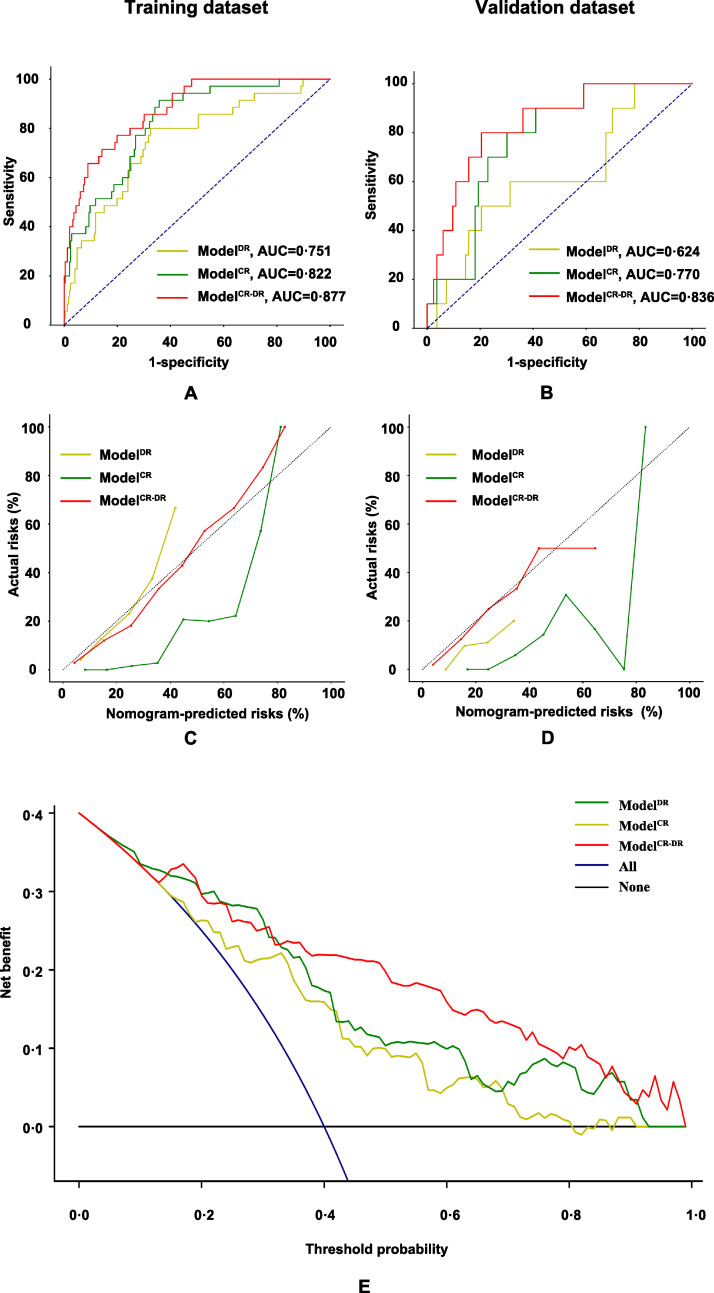
Comparison of the three models. The areas under the curve for Model^DR^, Model^CR^, and Model^CR-DR^ are 0•751, 0•822, 0•877, respectively, in the training dataset (A) and 0•624, 0•770, 0•836, respectively, in the validation dataset (B). Good calibrations (C and D) and decision curve (E) were also achieved.

**Fig. 5:**
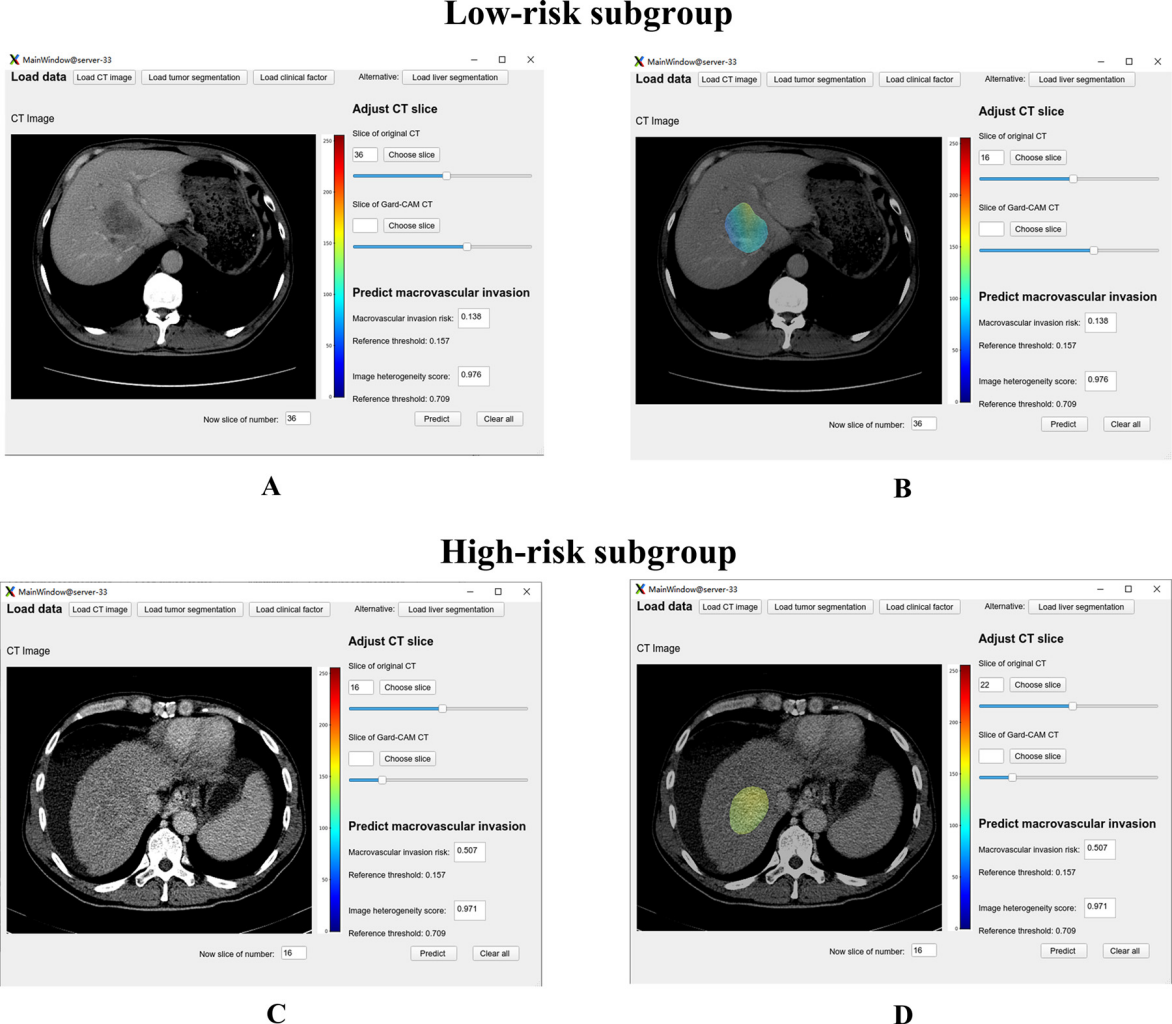
Patient examples by applet for Model^CR-DR^. (A, B) A 56-year-old male had single HCC with a maximum diameter of 54•4 mm and was initially treated by TACE. He was identified to be in the low-risk subgroup by our applet for Model^CR-DR^; no macrovascular invasion occurred for 1128 days and he remained alive at the end of the study. (C, D) A 68-year-old male had single HCC with a maximum diameter of 55•0 mm and was initially treated by TACE. He was identified to be in the high-risk subgroup by our applet for Model^CR-DR^; macrovascular invasion occurred after 245 days and death after 301 days.

Considering the three models (Model^DR^, Model^CR^, Model^CR-DR^), the AUCs were 0·751, 0·822, and 0·877, respectively, in the training dataset (Figure 4-A). The AUCs were 0·624, 0·770, and 0·836, respectively, in the validation dataset (Figure 4-B). The statistical analysis based on the Delong test, NRI, and IDI showed that Model^CR-DR^ performed better than Model^DR^ and Model^CR^ (Supplementary Table S2). Regarding the calibration, although Model^DR^ and Model^CR^ had an unsatisfactory performance, Model^CR-DR^ was better both in the training and validation datasets (Figure 4-C and 4-D). In the decision curve, Model^CR-DR^ also exhibited a better performance than Model^DR^ and Model^CR^ (Figure 4-E). Therefore, Model^CR-DR^ proved to be the optimal model. We constructed an applet accordingly (Figure 5, available at https://drive.google.com/drive/folders/1UdjobJ_zX3E-E4q6eUyBAWjWVutgM8PF?usp=sharing).

### 3.3 Subgroup and survival analysis using Model^CR-DR^

The subgroup analysis showed that most clinical factors had limited influence on the performance of Model^CR-DR^ (Supplementary Table S3), including age (*p* = 0·720; Supplementary Figure S2-A), sex (*p* = 0·421; Supplementary Figure S2-B), treatments (*p* = 0·853; Supplementary Figure S2-C), Child-Pugh class (*p* = 0·656; Supplementary Figure S2-E), BCLC stage (0 vs. A: *p* = 0·802; A vs. B, *p* = 0·714; 0 vs. B, *p* = 0·993; Supplementary Figure S2-F), max diameter (*p* = 0·533; Figure S2-G), number of lesions (*p* = 0·480; Supplementary Figure S2-H) and AFP level (<25 vs. 25–400: p = 0·065; <25 vs. >400, p = 0·373; 25–400 vs. >400, *p* = 0·243; Supplementary Figure S2-I). When patients were subdivided by radiologically confirmed cirrhosis, the two subgroups were statistically different (*p* = 0·030; Supplementary Figure S2-D). However, compared to the AUC of 0·836 in the validation dataset, the difference was primarily caused by an increase in the subgroup without cirrhosis (0·928), rather than a decrease in the subgroup with cirrhosis (0.823).

**Fig. 6:**
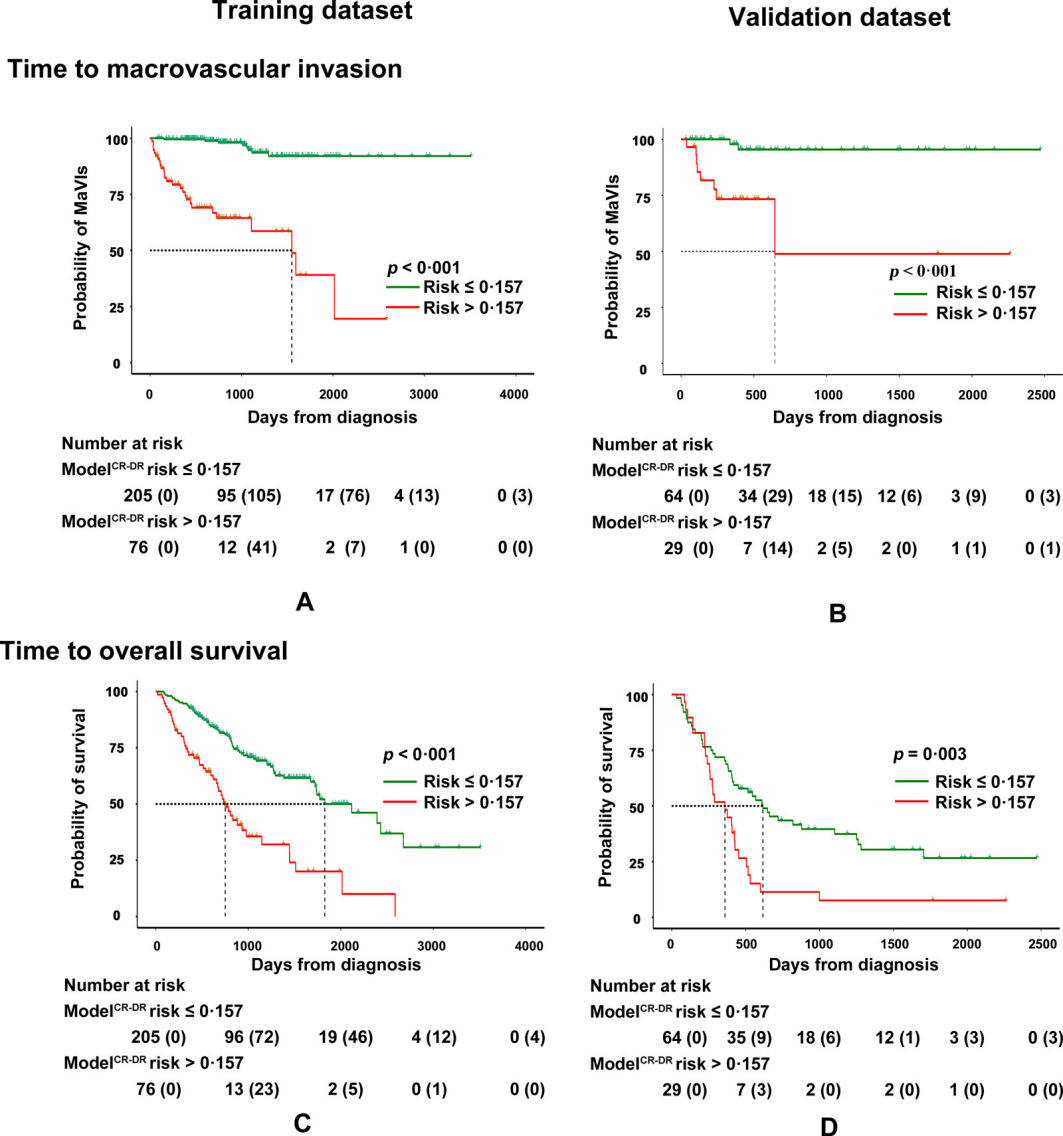
Survival analysis of Model^CR-DR^. Subdivided by Model^CR-DR^ risk at 0•157, the subgroup with a risk ≤ 0•157 and subgroup with a risk > 0•157 had statistical significance in times to macrovascular invasion (A and B) and overall survival (C and D) in both datasets.

The threshold of Model^CR-DR^ (0·157) was identified using the Youden index. Between the low-risk (risk ≤ 0·157) and high-risk subgroups (risk > 0·157), there were statistical differences in the time to macrovascular invasion in both the training (median: 1550 vs. infinite days; hazard ratio [HR] = 0·073; 95% confidence interval [CI]: 0·032–0·167; *p* < 0·001; Figure 6-A) and validation (median: 645 vs. infinite days; HR = 0·090; 95%CI: 0·022–0·366; *p* < 0·001; Figure 6-B) datasets. Regarding the OS, the two subgroups also had statistical differences in both the training (median: 360 vs. 617 days; HR = 0·344; 95%CI: 0·246–0·547; *p* < 0·001; Figure 6-C) and validation (median: 360 vs. 617 days; HR = 0·489; 95%CI: 0·279 – 0·859; *p* = 0·003; Figure 6-D) datasets.

### 3.4 Analysis of image heterogeneity using Model^CR-DR^

The median of the image heterogeneity scores within the tumor area (H-score) at 0·709 was also used to subdivide the patients. Between the low-risk (H-scores ≤ 0·709) and high-risk subgroups (H-score > 0·709), there were statistically significant differences in the time to macrovascular invasion in the training dataset (HR = 0·352; 95% CI: 0·181–0·688; *p* = 0·003; Supplementary Figure S3-A). Although there was a difference in the validation dataset, it was not statistically significant (HR = 0·195; 95%CI: 0·054–0·707; *p* = 0·082; Supplementary Figure S3-B). Considering the OS, the two subgroups showed statistical differences in both the training (HR = 0·417; 95%CI: 0·289–0·603; *p* < 0·001; Supplementary Figure S3-C) and validation (HR = 0.554; 95%CI: 0·345 – 0·892; *p* = 0·024; Supplementary Figure S3-D) datasets.

### 3.5 Comparison between Model^NO Seg-CR-DR^ and Model^CR-DR^

Without the subnetwork for segmentation, Model^NO Seg-CR-DR^ had a lower AUC than Model^CR-DR^ (Training: 0·828 vs. 0·877; Validation: 0·759 vs. 0·836; Supplementary Figure S4-A, S4-B). The difference was statistically significant per the Delong test in both the training and validation datasets (Supplementary Table S3). Furthermore, Model^NO Seg-CR-DR^ had worse calibration than Model^CR-DR^, especially in the validation dataset (Supplementary Figure S4-C, S4-D). The decreased performance in Model^NO Seg-CR-DR^ proved the necessity of the subnetwork for segmentation.

### 3.6 Comparison between Model^Logistic-CR-DR^ and Model^CR-DR^

With regard to the clinical and radiological factors, the multivariate logistic regression (backward: LR) identified the factors significantly related to the macrovascular invasion, including the BCLC stage, invasive shape, and anti-corona enhancement (Supplementary Table S4 and S5). Model^Logistic-CR-DR^ had a worse AUC than Model^CR-DR^ (training: 0·810 vs. 0·877; validation: 0·790 vs. 0·836; Supplementary Figure S5-A and S5-B). The difference was statistically significant per the Delong test and IDI in the training dataset (Supplementary Table S6). In addition, Model^Logistic-CR-DR^ showed a worse calibration than Model^CR-DR^ in both datasets (Supplementary Figure S5-C and S5-D). Based on the aforementioned results, an end-to-end deep learning algorithm was more appropriate for predicting future macrovascular invasion.

## 4 Discussion

Based on the multicenter database, this study used MTnet to construct a precise model (Model^CR-DR^) that could predict future macrovascular invasion in HCC. By combining information from the clinical/radiological factors and radiomics, Model^CR-DR^ achieved satisfactory results in terms of discrimination, calibration, and decision curve. Its performance was not influenced by the treatments or BCLC stage. Moreover, we used Model^CR-DR^ and H-score to subdivide the patients. Both high-risk subgroups had shorter times to macrovascular invasion and OS as predicted by the model. Model^CR-DR^ proved to be a precise and robust model that could be used to assist early intervention for macrovascular invasion in HCC.

Although macrovascular invasion can greatly jeopardize the survival of HCC patients, therapies can be used to treat it effectively [21,[25], [26]] Some of them may even be used to eliminate or control residual or radiologically occult tumor cells before it is too late [9]. Nonetheless, the lack of models for identifying high-risk populations limits the application of such early interventions. Considering this challenge, we may refer to valuable experience in the early prevention of distant metastasis in nasopharyngeal carcinoma, where a model was constructed to predict the risk of future distant metastasis [10]. To construct such a model with high accuracy, we assumed that besides the traditional clinical factors, data from medical images could be used to assess the heterogeneous information from the HCC lesions. Theoretically, although radiomics provides a massive amount of quantitative data, it may overlook the macro-changes across the tumor area, as well as differences adjacent to the outer boundary of tumor. Thus, we designed a set of radiological factors as a complementary assessment, either for morphological differences (such as fusion lesions or non-intact HCC capsule) or for changes outside the tumor boundary (such as corona and anti-corona enhancement). The better performance of Model^CR-DR^ compared to Model^DR^ and Model^CR^ proved that clinical/radiological factors and radiomic features were indispensable in achieving high accuracy in predicting the risks of future macrovascular invasion.

Deep learning algorithms have proven to be advantageous in constructing models for diagnosis and prognosis of cancers, especially for liver diseases [[16], [17], [18],^27^]. Meanwhile, among all the types of deep learning algorithms, multi-task learning combines severally related tasks during the training process and these can benefit from each other. Multi-task learning has attracted considerable attention in the field of medical image analysis [28], [29]; however, its application in HCC has been limited to microvascular invasion rather than macrovascular invasion [30]. Considering the potential advantages of multi-task learning, we constructed our MTnet to predict macrovascular invasion.

In the MTnet, the first challenge we faced was how to control the overfitting problem. Based on the potential function of multi-task learning in reducing the risk of overfitting [19]. we added tumor segmentation as a positive feedback for the analysis of the entire liver area. Compared to Model^NO Seg-CR-DR^, Model^CR-DR^ had better discrimination (per AUC with the Delong test) and calibration. The results show the necessity of a subnet for segmentation and prove the advantages of multi-task learning.

The other issue for the MTnet was how to screen the clinical/radiological factors. Although logistic regression is a conventional method, a deep learning algorithm may have advantages over logistic regression in highly complex model construction. Three factors were identified using the logistic regression. In addition to the BCLC stages, an invasive shape was designed to assess the ability of the tumor to invade beyond the limitations of surrounding tissues, whereas anti-corona enhancement was designed to assess the abnormal area of low attenuation around the tumor. Both radiological characteristics reflected the influence of the surrounding area by the HCC, which contained structures of the blood vessels. However, compared to Model^Logistic-CR-DR^, the discrimination improvement of Model^CR-DR^ was only significant in the training dataset. The calibration was obvious in both datasets. The results proved the rationality of screening clinical/radiological factors using a deep learning algorithm.

This study has some limitations. First, the strict inclusion criteria limit the sample size of this study. Considering the complexity of HCC management, although we prove that treatments and BCLC stages have limited influence on the robustness of our model, the conclusions should be tested on a larger sample size in the future. With the enlargement of sample size, we may use extra validation datasets to further confirm the robustness of our model. We may also perform a more detailed subgroup analysis, such as anatomical vs. non-anatomical resection, drug-eluting bead vs conventional TACE, etc. Second, this study was based on a Chinese population, where most of the HCC cases were caused by hepatitis B. Therefore, for western populations (where HCC is mainly caused by hepatitis C or alcoholic hepatitis), our model needs further validation. Third, to further improve the performance, the combination of other data resources such as MRI or pathology may be explored. Fourth, owing to the "black box" effect, which is common in deep learning studies, we could not provide pathological interpretation for the deep learning radiomics. Although our results have demonstrated the stability of our final model, further research may be required to explore its relationship with pathological changes in HCC.

In conclusion, by combining clinical factors, radiological characteristics, and radiomics, our multi-task deep learning network-based model successfully predicted future macrovascular invasion in the HCC. Considering high-risk populations, besides the current guideline-recommended treatments, more therapies such as a combination of targeted therapy and/or immunotherapy should be explored to perform timely prevention or early treatments and increase the survival rate.

## Contributors

Sirui Fu, Haoran Lai, and Qiyang Li conceived and designed the project with supervision from Ligong Lu and Meiyan Huang. Sirui Fu, Haoran Lai and Meiyan Huang accessed the raw data and were responsible for the accuracy. Jianwen Huang, Xiaoqun Li, Xiaofeng He and Tao Wang acquired the data. Yao Liu and Jianfeng Yan segmented the data manually. Haoran Lai, Jiawei Zhang and Xiumei Chen provided in the statistical analysis. All authors were involved in drafting and technical support in deep learning methods. Chongyang Duan assisted in statistical analysis. All authors were involved in drafting and reviewing the manuscript and approved the final manuscript for submission.

## Data sharing statement

Due to the privacy of patients, the data related to patients cannot be available for public access but can be obtained from the corresponding author on reasonable request approved by the institutional review board of Zhuhai People's Hospital

## Declaration of Competing Interest

The authors have no conflicts of interest to declare.

## Appendix Supplementary materials

Image, application 1

Image, application 2
