# Supplementary material for: Multi-task deep learning network to predict future macrovascular invasion in hepatocellular carcinoma
Source: eClinicalMedicine. 2021 Dec 9;42:101201. doi: 10.1016/j.eclinm.2021.101201 (PMC8668827; doi:10.1016/j.eclinm.2021.101201)
Supplement: Supplementary file 1 [file mmc1.docx]

Caption for supplementary material

Su**pplementary Table S1.** Computed tomography parameters at the participating hospitals

Supplementary Text S1. First block for information extracted from the CT images (Figure 2-A and 2-B).

Supplementary Text S2. Second block for information from clinical factors and radiological characteristics (Figure 2-C).

Supplementary Text S3. Third block to predict the risk of macrovascular invasion.

Supplementary Text S4: Loss function

Supplementary Text S5: Image heterogeneity score of the tumor area.

Supplementary Figure S1. Schematic diagram for the calculation of image heterogeneity score of the tumor area. (A) Extraction of minimum and maximum values in the color map for the minmax operator. (B) Extraction of minimum and maximum values in the normalized color map within the tumor area.

Table S2. Comparison of Model^DR^, Model^CR^, and Model^CR-DR^ (*p* values)

**Supplementary Figure S2. Subgroup analysis.** With the exception that patient without radiological confirmed cirrhosis showed higher AUC (D), there were no statistical difference between subgroups regarding age (A), sex (B), treatments (C), Child-Pugh class (E), BCLC stage (F), max diameter (G), number of lesions (H), and AFP level (I).

**Supplementary Figure S3. Survival analysis by H-score.** When subdivided by the median of H-score (0·709), significant differences were observed between the two subgroups in the time to macrovascular invasion in the training dataset (A), whereas the validation dataset did not achieve statistical significance (B). Considering the overall survival, the two subgroups had a statistical difference in both the training (C) and validation (D) datasets.

**Supplementary Figure S4: Comparison between Model^NO Seg-CR-DR^ and Model^CR-DR^.** Without the subnetwork for segmentation, Model^NO Seg-CR-DR^ had a lower AUC than Model^CR-DR^ in both the training (A) and validation (B) datasets. In addition, Model^NO Seg-CR-DR^ had a worse calibration than Model^CR-DR^ in both the training (C) and validation (D) datasets.

Su**pplementary** Table S3. Comparison between Model^NO Seg-CR-DR^ and Model^CR-DR^

Su**pplementary** Table S4. Univariate logistic regression analysis of clinical and radiological factors

Su**pplementary** Table S5. Multivariate logistic regression analysis of clinical and radiological factors

**Supplementary Figure S5: Comparison between Model^Logistic-CR-DR^ and Model^CR-DR^.** Without the clinical and radiological factors screened by logistic regression, Model^Logistic-CR-DR^ had a lower AUC than Model^CR-DR^ in both the training (A) and validation (B) datasets. In addition, Model^Logistic-CR-DR^ had a worse calibration than Model^CR-DR^ in both the training (C) and validation (D) datasets.

Su**pplementary** Table S6. Comparison between Model^CR-DR -Logistic^ and Model^CR-DR^
