# Supplementary material for: Multi-task deep learning network to predict future macrovascular invasion in hepatocellular carcinoma
Source: eClinicalMedicine. 2021 Dec 9;42:101201. doi: 10.1016/j.eclinm.2021.101201 (PMC8668827; doi:10.1016/j.eclinm.2021.101201)
Supplement: Supplementary file 2 [file mmc2.docx]

| **Table S1.** Computed tomography parameters at the participating hospitals | | | | | |
| --- | --- | --- | --- | --- | --- |
|  | Training | | | | Validation |
|  | NFH | SPH | YPH | ZPH | ZCPH |
| Scanner | PB | PB | PB | SSDF | PB |
| TV (kV) | 120 | 120 | 120 | 120 | 120 |
| TC (mA) | 142 | 250 | 300 | 160 | 250 |
| RT (s) | 0·75 | 0·5 | 0·75 | 0·5 | 0·5 |
| DC (mm) | 128×0·625 | 64×0·625 | 64×0·625 | 64×0·625 | 128×0·625 |
| FOV (mm) | 300×300 | 500×500 | 350×350 | 350×350 | 350×350 |
| PM | 512×512 | 1024×1024 | 512×512 | 512×512 | 512×512 |
| Reconstruction | FSH (C) | FSH (C) | FSH (C) | FSH (C) | FST (B) |
| ST (mm) | 5 | 5 | 2 | 2/ 5 | 5 |
| PB: Philips Brilliance; SSDF: Siemens Somatom Definition Flash; TK: tube voltage; TC: tube current; RT: rotation time; DC: detector collimation; FOV: field of view; PM: pixel matrix; FSH: filter sharp; FST: filter standard; ST: slice thickness. | | | | | |

Supplementary Text S1. First block for information extracted from the CT images (Figure 2-A and 2-B).

To eliminate the disturbance caused by irrelevant information, the areas in the CT images with attenuations less than −17 HU were set as −17 HU, and those larger than 201 HU were set as 201 HU. Thereafter, a pre-trained model constructed with “no new Unet” was applied to segment the liver.^1^ Subsequently, two independent radiologists checked the liver segmentation and made necessary corrections. Therefore, only the liver and tumor remained in the processed CT images. Finally, all the CT images were resized to 160×160×160, using bilinear interpolation for a uniform input size of the network.

Considering these procedures, a modified Unet, including an encoder (Figure 2-A) and a decoder (Figure 2-B), was used for the segmentation task. The encoder was applied to extract useful image information, and the decoder was used to segment the tumors. Theoretically, macrovascular invasion might be more related to tumor lesions, making the tumor area more important. Therefore, the decoder was used as a positive feedback to the encoder through tumor segmentation to ensure that the encoder focused on the tumor area. Obtaining this positive feedback, rich image information can be provided by the encoder.

Supplementary Text S2. Second block for information from clinical factors and radiological characteristics (Figure 2-C).

Regarding the clinical and radiological data, continuous variables were processed by standardization as follows:

$$x^{'}=\frac{x-\mu}{\sigma}$$

where *x* is a continuous variable; $\mu$ and $\sigma$ represent the mean and variance of the continuous variable, respectively. The $\mu$ and $\sigma$ values were obtained from the training set and applied to the validation set. For discrete variables, the original data were included directly.

The discrete features were used as input to be fed into a word embedding layer,^2^ which can learn the continuous expression of the features in the training stage. Thereafter, the new continuous and original continuous features were concatenated and fed into two fully connected layers to extract potential information regarding the clinical and radiological factors.

Supplementary Text S3. Third block to predict the risk of macrovascular invasion.

The potential information extracted from the first and second blocks was concatenated as input to a two-layer fully connected network with a dropout layer^3^ to construct a combined model for classification. The entire network was implemented end-to-end.

Supplementary Text S4: Loss function

Considering the classification loss, the weight cross-entropy loss function was applied to alleviate the class imbalance problem. This can be defined as

$$L_{ce}=\frac{1}{N}\sum_{i} -[\alpha y_{i}\cdot\log(p_{i})+(1-\alpha)(1-y_{i})\cdot\log(1-p_{i})]$$

where $y_{i}$ is the class label of sample *i*, *N* is the number of patients, $p_{i}$ is the prediction probability of sample *i*; $\alpha$ is the balance coefficient, which is set to 0.85. Regarding the segmentation loss, the dice loss function was used. This is expressed as

$$L_{dice}=1-\frac{2}{\left|K\right|}\sum_{k\in K} \frac{\sum_{i} \mu_{i}^{k}\upsilon_{i}^{k}}{\sum_{i} \mu_{i}^{k}+\sum_{i} \upsilon_{i}^{k}}$$

where $\mu$ is the softmax output of the segmentation network, $\upsilon$ is a one-hot encoding of the ground truth segmentation map, *i* is the voxel number in the input image, and *K* is the number of segmentation classes. Therefore, the total loss function is defined as follows:

In the training stage, a stochastic gradient descent algorithm^4^ was implemented to optimize the total loss function.

Supplementary Text S5: Image heterogeneity score of the tumor area.

The color map was firstly normalized to the range of [0, 1] by using a minmax operator, which was listed in Eq. (5). Then, the minimum and maximum values within the tumor area were extracted and the image heterogeneity score (H-score) of tumor area can be calculated by using Eq. (6).

$$x^{'}=\left(x-x_{\min}\right)/\left(x_{\max}-x_{\min}\right)$$

$$\text{H-score}=x_{t\max}^{'}-x_{t\min}^{'}$$

where *x* is the value in color map, $x_{\min}$ and $x_{\max}$ are the minimum and maximum values in color map, respectively. The $x_{t\min}^{'}$ and $x_{t\max}^{'}$ are the minimum and maximum values in normalized color map within tumor area, respectively.

**
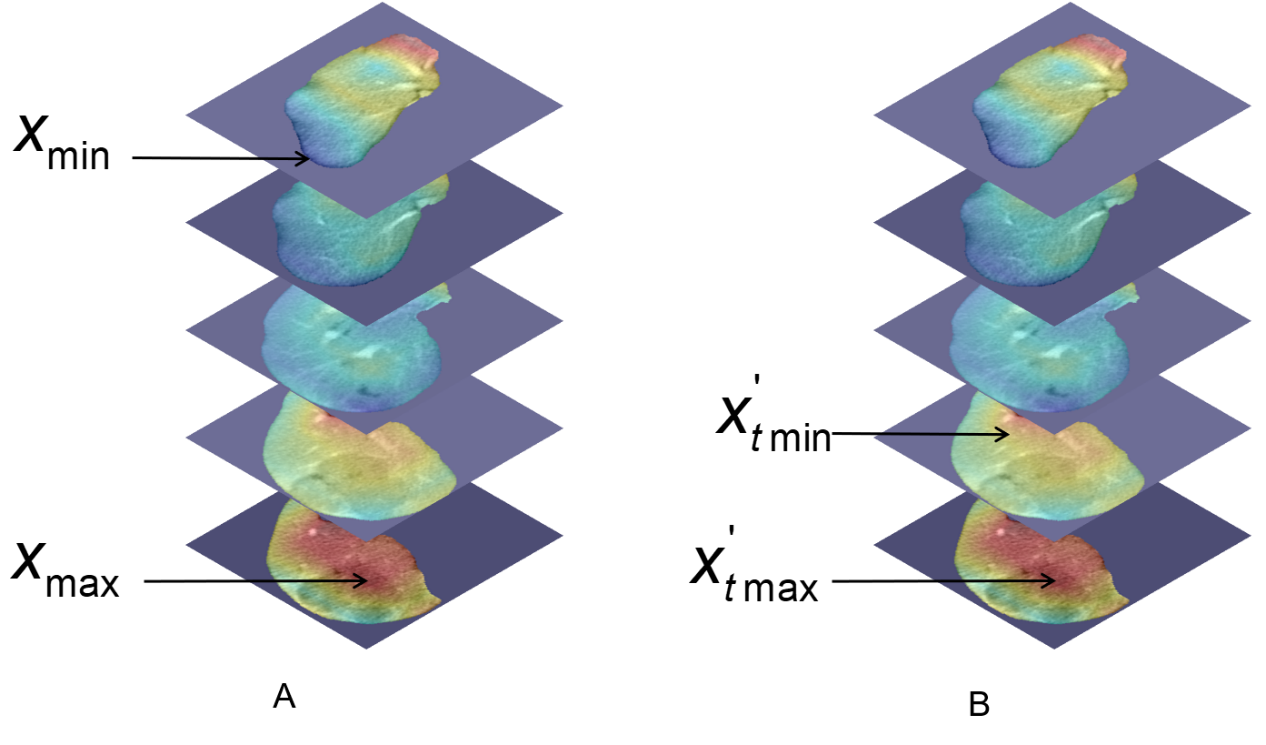
**

Supplementary Figure S1. Schematic diagram for the calculation of image heterogeneity score of the tumor area. (A) Extraction of minimum and maximum values in the color map for the minmax operator. (B) Extraction of minimum and maximum values in the normalized color map within the tumor area.

| Table S2. Comparison of Model^DR^, Model^CR^, and Model^CR-DR^ (*p* values) | | | | | | |
| --- | --- | --- | --- | --- | --- | --- |
|  | Training dataset | | | Validation dataset | | |
|  | Delong | NRI | IDI | Delong | NRI | IDI |
| Model^DR^ vs. Model^CR^ | 0·179 | 0·413 | <0·001^*^ | 0·209 | 0·148 | 0·046^*^ |
| Model^CR-DR^ vs. Model^CR^ | 0·047^*^ | 0·847 | 0·001^*^ | 0·040^*^ | 0·007^*^ | 0·769 |
| Model^CR-DR^ vs. Model^DR^ | 0·013^*^ | 0·341 | <0·001^*^ | 0·047^*^ | 0·028^*^ | 0·062 |
| ^*^With statistical difference  NRI: net reclassification improvement; IDI: integrated discrimination improvement | | | | | | |


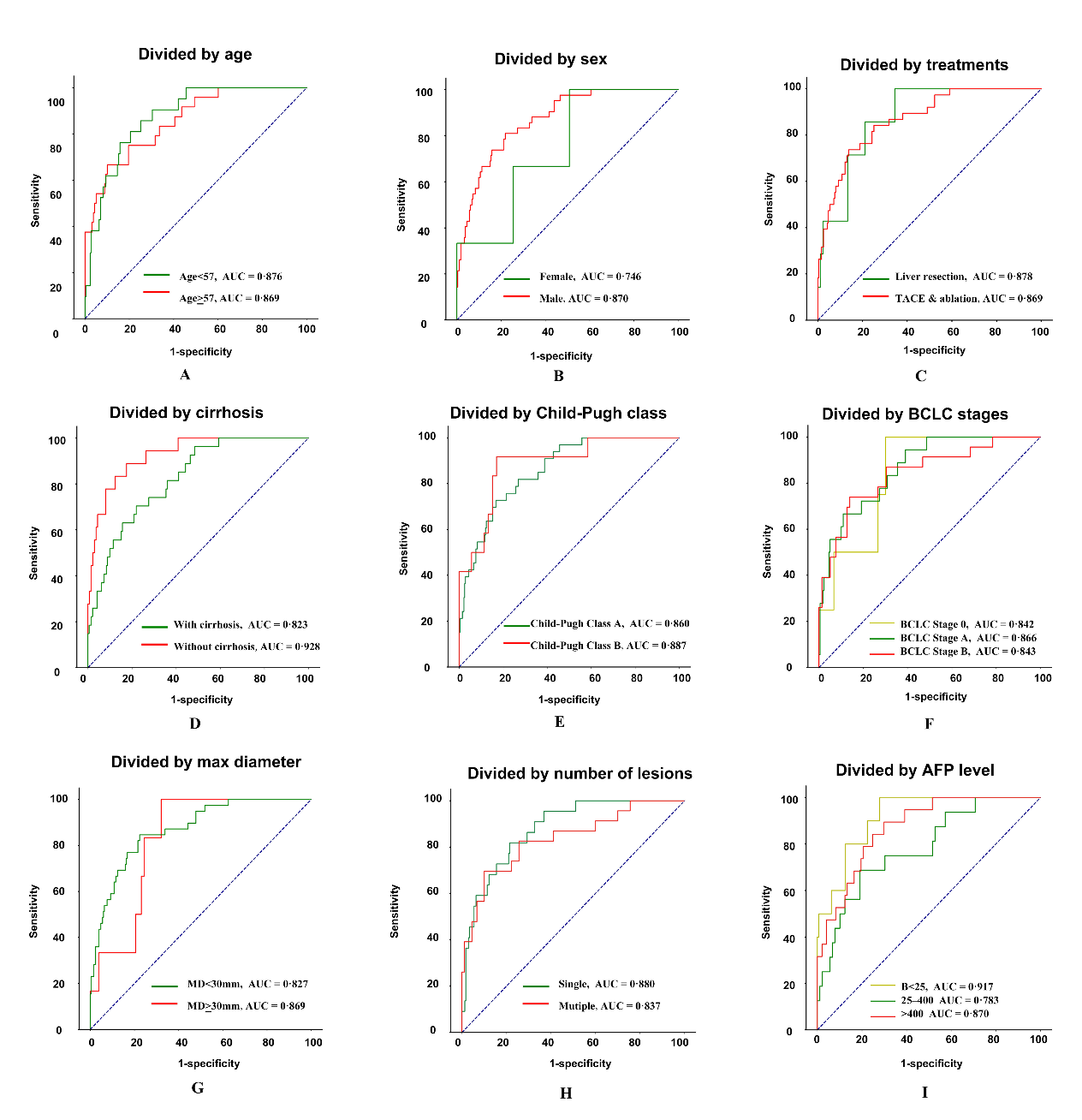


Supplementary Figure S2. Subgroup analysis. With the exception that patient without radiological confirmed cirrhosis showed higher AUC (D), there were no statistical difference between subgroups regarding age (A), sex (B), treatments (C), Child-Pugh class (E), BCLC stage (F), max diameter (G), number of lesions (H), and AFP level (I).

**
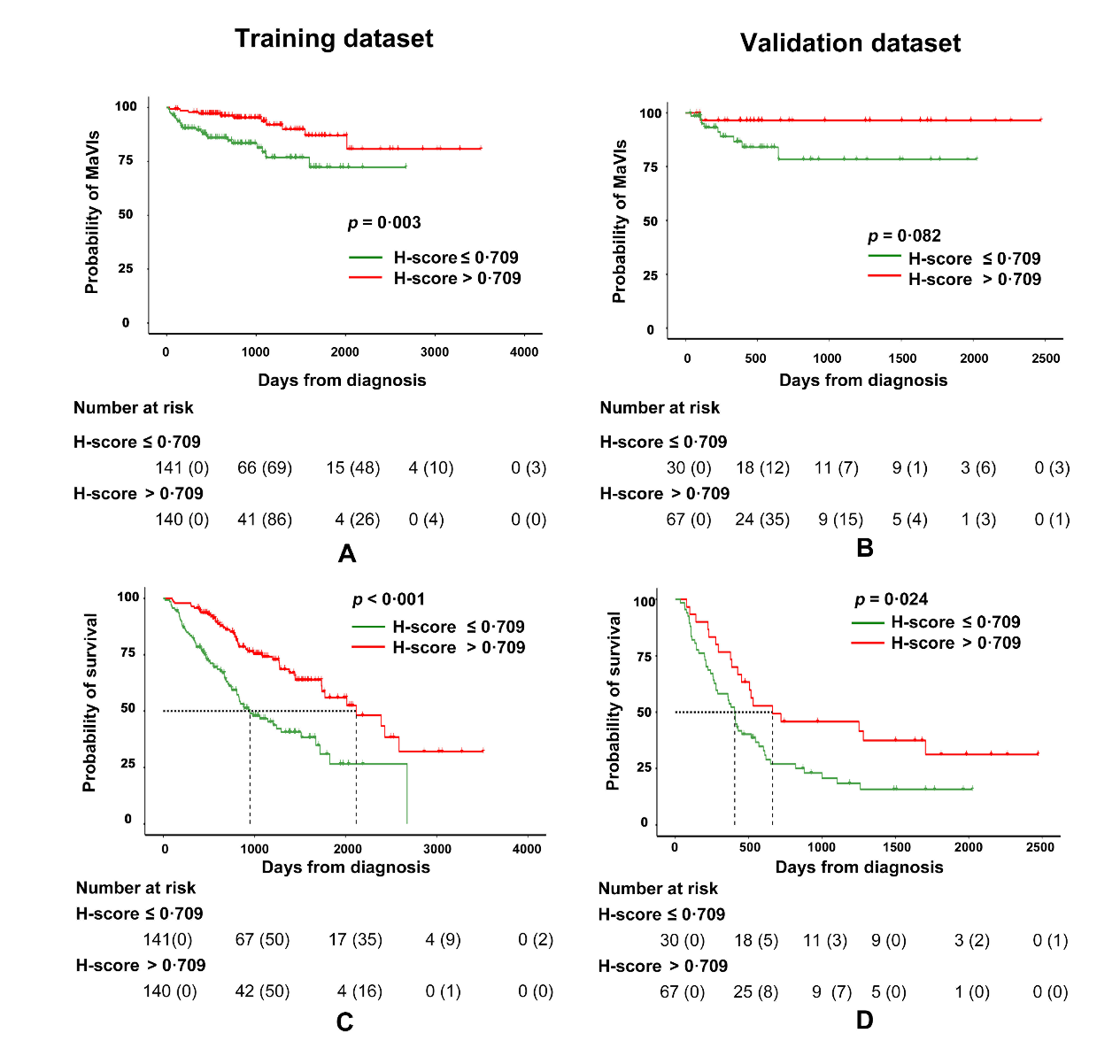
**

Supplementary Figure S3. Survival analysis by H-score. When subdivided by the median of H-score (0·709), significant differences were observed between the two subgroups in the time to macrovascular invasion in the training dataset (A), whereas the validation dataset did not achieve statistical significance (B). Considering the overall survival, the two subgroups had a statistical difference in both the training (C) and validation (D) datasets.


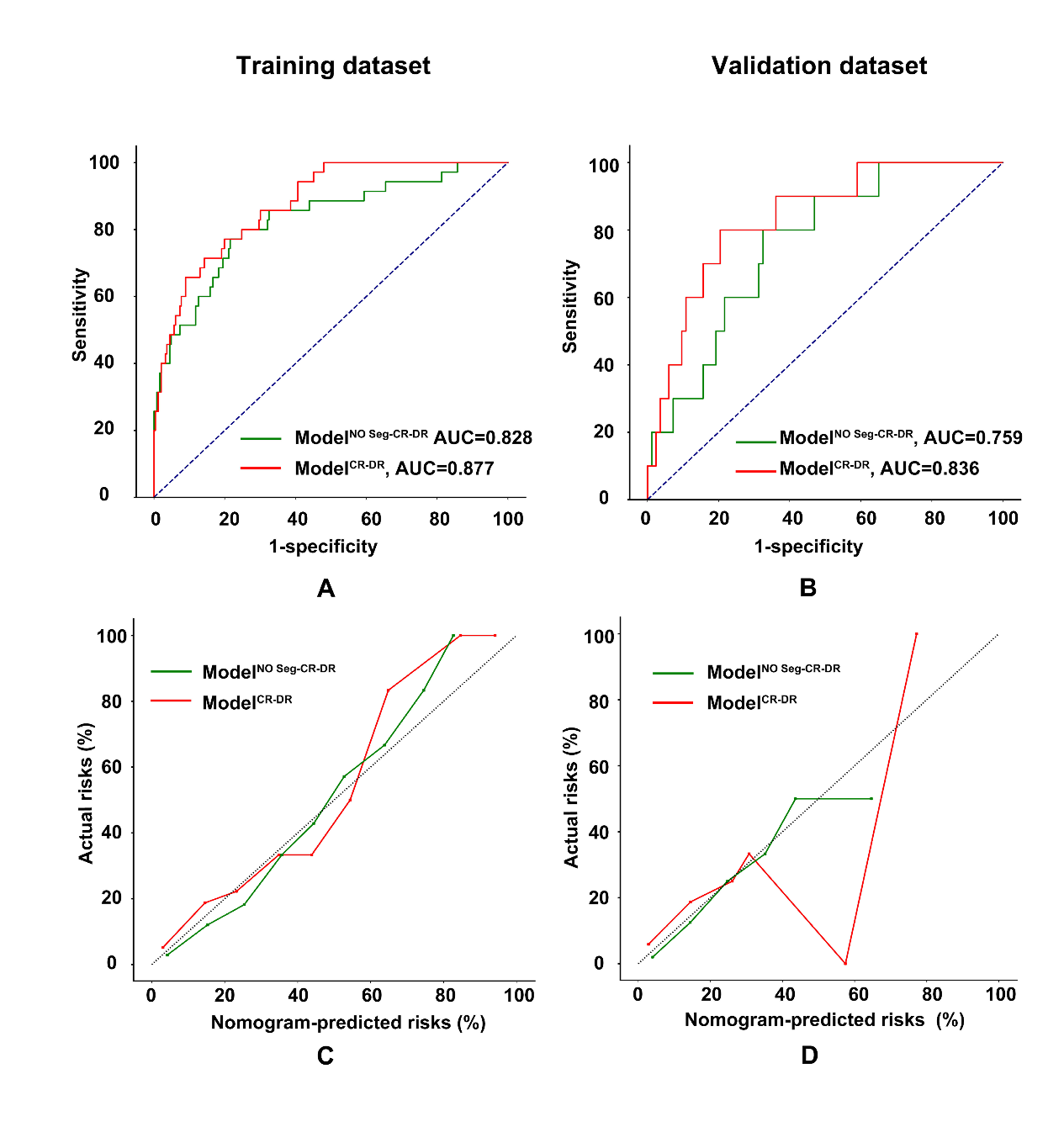


Supplementary Figure S4: Comparison between Model^NO Seg-CR-DR^ and Model^CR-DR^. Without the subnetwork for segmentation, Model^NO Seg-CR-DR^ had a lower AUC than Model^CR-DR^ in both the training (A) and validation (B) datasets. In addition, Model^NO Seg-CR-DR^ had a worse calibration than Model^CR-DR^ in both the training (C) and validation (D) datasets.

| Table S3. Comparison between Model^NO Seg-CR-DR^ and Model^CR-DR^ | | |
| --- | --- | --- |
|  | Training dataset | Validation dataset |
| Delong test | 0·032^*^ | 0·032^*^ |
| NRI | 0·824 | 0·152 |
| IDI | 0·353 | 0·059 |
| ^*^With statistical difference  NRI: net reclassification improvement; IDI: integrated discrimination improvement | | |

| Table S4. Univariate logistic regression analysis of clinical and radiological factors | | |
| --- | --- | --- |
| Factors | OR (95% CI) | *p*-value |
| Clinical factors |  |  |
| Sex | 0·443 (0·103–1·311) | 0·193 |
| Age | 1·018 (0·989–1·049) | 0·228 |
| BCLC stage* |  |  |
| 0 |  | Reference |
| A | 0·492 (0·152–1·588) | 0·236 |
| B | 0·287 (0·133–0·621) | 0·002 |
| Child-Pugh class | 1·895 (0·787–4·238) | 0·133 |
| Treatments |  |  |
| Liver resection |  | Reference |
| TACE | 1·33×10^8^ (<0·001–NA) | 0·999 |
| Ablation | 2·81×10^8^ (<0·001–NA) | 0·999 |
| HBV | 3·009 (0·596–54·859) | 0·290 |
| AFP (ng/mL) * |  |  |
| <25 |  | Reference |
| 25–400 | 0·388 (0·159–0·945) | 0·037 |
| >400 | 0·845 (0·364–1·959) | 0·694 |
| Number of lesions* |  |  |
| 1 |  | Reference |
| 2 | 0·211 (0·080–0·561) | 0·002 |
| 3 | 0·319 (0·096–1·059) | 0·062 |
| >3 | 0·472 (0·120–1·860) | 0·283 |
| Maximum diameter | 1·005 (0·997–1·014) | 0·223 |
| Cirrhosis | 0·993 (0·487–2·061) | 0·984 |
| Radiological factors |  |  |
| Location | 1·394 (0·923–2·066) | 0·104 |
| Adjacent to liver surface | 1·100 (0·356–4·821) | 0·882 |
| Fusion lesion | 1·696 (0·826–3·469) | 0·146 |
| Invasive shape* | 2·389 (1·164–5·084) | <0·019 |
| HCC capsule* |  |  |
| Absent |  | Reference |
| Non-intact | 4·533 (0·915–22·465) | 0·064 |
| Intact | 4·250 (0·972–18·575) | 0·055 |
| HCC capsule breakthrough | 2·019 (0·836–4·533) | 0·010 |
| Corona enhancement | 2·411 (1·028–5·338) | 0·121 |
| Anti-corona enhancement* | 1·791 (0·859–3·733) | 0·035 |
| Mosaic architecture | 1·206 (0·523–3·130) | 0·676 |
| Nodule-in-nodule architecture | 1·206 (0·526–3·130) | 0·103 |
| HCC enhancement ratio |  |  |
| <25% |  | Reference |
| 25–50% | 1·619 (0·544–4·819) | 0·387 |
| 50–75% | 1·587 (0·605–4·160) | 0·345 |
| >75% | 1·727 (0·685–4·356) | 0·247 |
| *Factors with a *p* < 0·100 were included for the multivariate regression.  AFP: alpha-fetoprotein; BCLC: BCLC: Barcelona Clinic Liver Cancer; CI: confidence interval; HBV: hepatitis B virus; HCC: hepatocellular carcinoma; OR: odds ratio. | | |

| Table S5. Multivariate logistic regression analysis of clinical and radiological factors | | |
| --- | --- | --- |
| Factors | OR (95% CI) | *p*-value |
| Clinical factors |  |  |
| BCLC stage |  |  |
| 0 |  | Reference |
| A | 0·871 (0·242–3·132) | 0·832 |
| B | 0·355 (0·160–0·788) | 0·011* |
| Radiological factors |  |  |
| Invasive shape | 2·081 (0·950–4·733) | 0·071 |
| Anti-corona enhancement | 2·158 (0·882–5·010) | 0·080 |
| *Factors with a *p* < 0·050  BCLC: Barcelona Clinic Liver Cancer; CI: confidence interval; OR: odds ratio. | | |


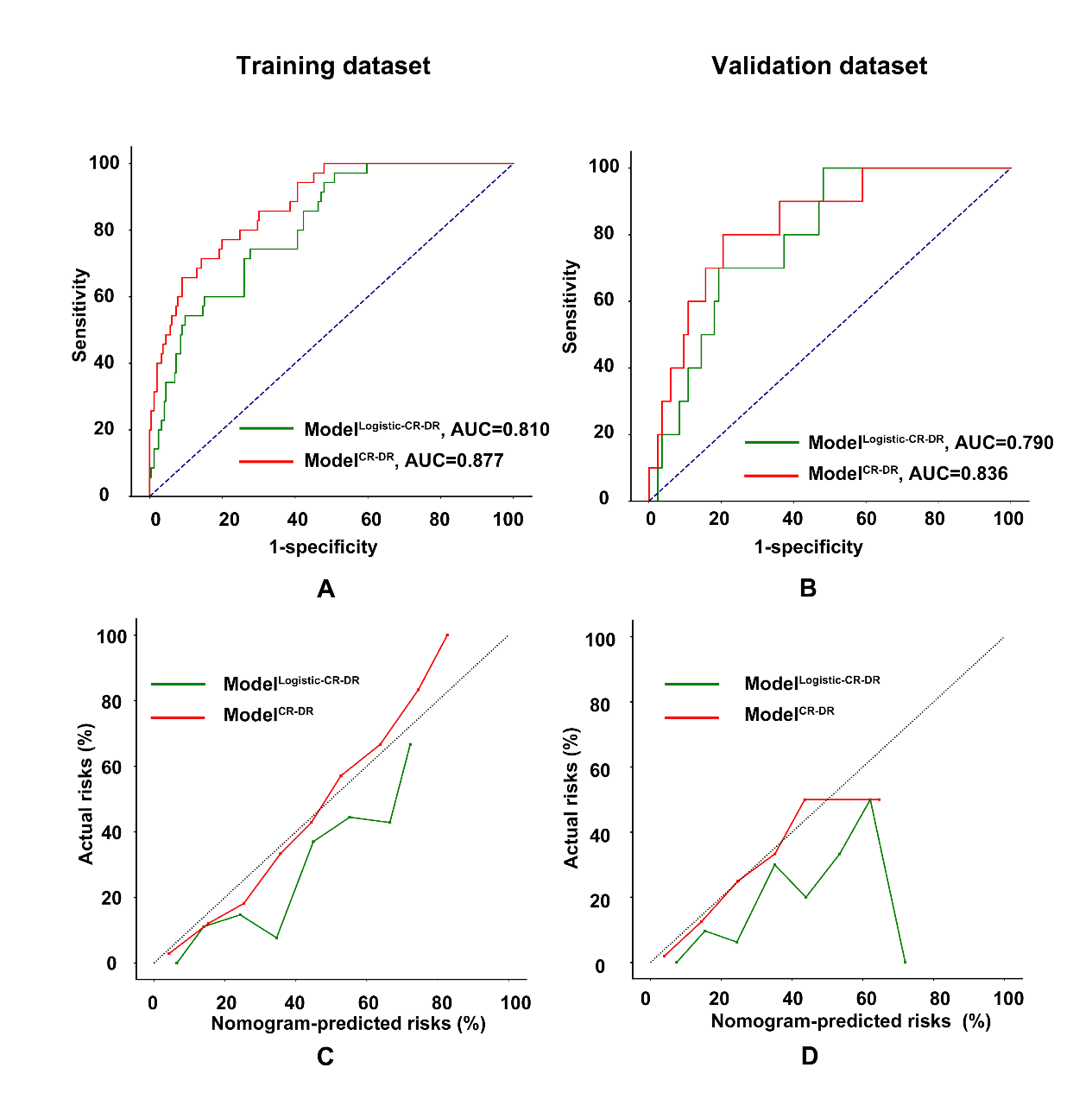


Supplementary Figure S5: Comparison between Model^Logistic-CR-DR^ and Model^CR-DR^. Without the clinical and radiological factors screened by logistic regression, Model^Logistic-CR-DR^ had a lower AUC than Model^CR-DR^ in both the training (A) and validation (B) datasets. In addition, Model^Logistic-CR-DR^ had a worse calibration than Model^CR-DR^ in both the training (C) and validation (D) datasets.

| Table S6. Comparison between Model^CR-DR -Logistic^ and Model^CR-DR^ | | |
| --- | --- | --- |
|  | Training dataset | Validation dataset |
| Delong test | 0·011^*^ | 0·458 |
| NRI | 0·126 | 0·412 |
| IDI | <0·001^*^ | 0·723 |
| ^*^With statistical difference  NRI: net reclassification improvement; IDI: integrated discrimination improvement | | |

References:

1. Isensee F, Jaeger P F, Kohl S, Petersen J, Maier-Hein K H. "nnU-Net: a self-configuring method for deep learning-based biomedical image segmentation." *Nature Methods*, 2020, pp. 1-9.

2. Goldberg Y, Levy O. "word2vec Explained: deriving Mikolov et al.'s negative-sampling word-embedding method," arXiv preprint arXiv:1402.3722, 2014.

3. Baldi P, Sadowski P J J A. "Understanding dropout," *Advances in neural information processing systems*, 2013, vol. 26, pp. 2814-2822.

4. Bottou L. "Large-scale machine learning with stochastic gradient descent," in Proceedings of COMPSTAT'2010: Springer, 2010, pp. 177-186.
